# Progress of Veterinary Science

**Published:** 1882-04

**Authors:** 


### PROGRESS OF VETERINARY SCIENCE.

---

The Best Disinfecting Agents, according to Mr. W. M. Hamlet, are in general those capable of exerting an immediate and powerful oxidizing action, and that it is active oxygen, whether from the action of chlorine, nitric oxide, or hydrogen peroxide, which must be regarded as the greatest known enemy to bacterial life.

Resuscitation of Frozen Animals.—Out of twenty animals treated by Prof. Manassen, by the method of gradual resuscitation in a cold room, fourteen died; of twenty introduced at once into a warm apartment, eight perished; while of twenty placed immediately in a hop bath all recovered. —*International Surg. Record.*

Milk Vein.—What is usually called the milk vein of a cow is the vein which conveys the blood passing through the udder along the belly to the heart. Its size should always be noted, as it indicates the amount of blood passing through the udder which, in a great measure, governs the amount of milk.

Oil Meals.—According to Prof. Armsby, of the Conneticut Experiment Station, cottonseed meal is the more valuable for fattening animals and for cows in milk, while linseed meal is better for growing stock, having less "fat" and more "nitrogen free extract." The exact proportionate value has not been ascertained, as no long-continued experiments in actual feeding have been made. Cottonseed meal contains 42.9 per cent. of "protein" compounds, 17.3 per cent. of fat, 18.6 per cent of nitrogen free extract, and 7.4 per cent. of crude fibre; dry matter, 93 per cent. Linseed meal has 29.4 per cent. of protein compounds, 11.8 of fat, 34.1 of nitrogen free extract, and 8.8 of crude fibre; dry matter, 90.6 per cent. These are the averages of the analyses in Prof. Armsby's tables.—*Country Gentleman.*

Pleuro-Pneumonia in Cattle.—The Queens County (N. Y.) Farmers' and Dairymen's Association held a special meeting for the purpose of hearing the report of the committee appointed by the association to examine into the question of inoculation to prevent the spread of pleuro-pneumonia, and also to investigate the manner in which one of the State veterinary surgeons had or had not complied with the laws on the subject, passed in 1878 and 1879. The committee reported that inoculation of neat cattle is the only preventive against the spread of pleuro-pneumonia. The committee also reported that Alexander O. Hopkins, State Veterinary Surgeon, had violated the laws of 1878 and 1879, and instructions under the same, in that he had permitted infected cattle, killed to prevent the spread of the disease, to be dressed by butchers and shipped to the New York market. The committee reported that they had waited upon Governor Cornell, and informed him of these facts.

THE EFFECTS OF COD-LIVER OIL ON MONKEYS.—In August, 1880, a large ourang-outang was placed in the Westminster Aquarium. He suffered from a very severe bronchitis, contracted during the intense cold of the winter. Treatment consisted in keeping the air moist by means of steam and by giving internally cod-liver oil, milk, and a small quantity of carbonate of ammonia. He recovered completely under the treatment and is now in perfect health, but nevertheless the cod-liver oil is continued, the animal using a wine-bottle full a week.

The reporter of the observation observes that we must attribute the return to health to the cod-liver oil. In the zoölogical gardens of Frankfort, where the climate is not favorable to the monkey tribe, they have examples of great longevity in these animals. Mandrils, chacmas, and other animals of this class, live in this establishment fifteen to eighteen years, whereas the average duration of life in other institutions of the kind is about two years. The remarkably good health and long life of the quadrumina in these gardens is attributed to the fact that the director, Dr. Schmitt, gives them all cod-liver oil during the winter season. These observations seem to show that the effect of cod-liver oil is essentially the same on the monkeys and apes as on human beings.—*L'Acclamation.*

THE VIVISECTION QUESTION IN THE GERMAN PARLIAMENT.—A petition was presented to the German Parliament some time ago, asking for the restriction or abolition of vivisection. The matter was referred to a committee, which, through its chairman, Prof. Hüter, of Griefswald, has recently reported against the petition. The report was accompanied with a motion that, considering (1) that, in the interest of science, vivisection appears to be indispensable in teaching institutions; (2) that changes in the penal statutes, in the direction desired by the petitioners, have not been shown to be necessary; (3) that the petitioners have the opportunity of laying their complaints of any abuses in regard to vivisection before the local authorities who have the regulation of the teaching institutions. Prof. Virchow denied the correctness of the statement made by the petitioners, that experiments were performed in the universities to a great extent by students. He proceeded to show that vivisection was indispensable to the progress of medical science.—*Medical Record.*

A SIMPLE MEANS OF DETECTING TRICHINÆ.—According to John Phin the parts of an animal that should first be examined are the diaphragm, tenderloin, and muscles about the head and throat. In a ham the most likely place is that part at which the muscle ends in a tendon. Cut off a thin slice with a very sharp knife, or with a pair of scissors curved on the flat. This thin section should then be soaked for some minutes in acetic acid, spread out on a thin piece of glass, and covered with another similar piece. These two slips are then pressed together. A compressorium, by means of which the plates of glass are forced together by a lever and screw, answers admirably. But better still he finds the trichinoscope, which is a compressorium holding the two glass strips, but fitted with a simple microscope on a sliding frame, which permits the examination of a specimen in each part. His plan is as follows: A thin piece of flesh, moistened with a mixture of equal parts of acetic acid and glycerine, is placed on the lower plate and spread by means of needles fixed in wooden handles. The upper plate is then brought down on the lower one, and the screw is turned into the slot in which it fits. By turning the nut, any degree of pressure may be brought to bear on the flesh, which is thus rendered so thin and transparent that any trichinæ present will be readily brought into view.—*Medical Record.*

NOTES ON SEVEN FATAL CASES OF HYDROPHOBIA.—Mr. Southam has tabulated his notes of all the cases of hydrophobia seen during two years' term of office in the Manchester Royal Infirmary, and furnishes some items of interest from a clinical point of view. As the treatment cannot be based upon any definite pathological condition which is to be met, it became purely symptomatic, and resolved itself into four principal methods: 1st, by chloral and opium; 2d, by chloroform and curara; 3d, by tracheotomy; 4th, by the hot air bath. Of the four drugs mentioned, chloral appeared to secure the most beneficial results, prolonging life, and temporarily arresting the spasms. Its administration was readily effected hypodermically, the introduction of the needle causing no spasm. With regard to curara, the author found in two instances that there were alarming symptoms of respiratory weakness, once after one-sixth grain had been administered, and yet the spasms were not relieved. Tracheotomy, which was performed to obviate death from spasms of the glottis, was of little use. In six of the cases death was due to gradual heart-failure, and in only one to spasm of the glottis. In respect to the temperature, it was found in three cases to have risen above 103° F., and in one to above 105° F. The urine of all the cases contained albumen. In three instances there was sugar in the urine, indicating, probably, that some abnormal condition of the medulla oblongata was present.—*Medical Record.*

HYDROPHOBIA: ITS SUCCESSFUL TREATMENT.—Mr. Ruxton, a surgeon in the East Indies, reports a very remarkable case, which seems worthy of being classed with the small number of cures that are now on record. A boy, between five and six years of age, was bitten in 1874 by a bull-bitch, that was subsequently killed. The bites were deep and severe, but were freely cauterized with fuming nitric acid, causing considerable loss of tissue. Carbolized oil was subsequently employed as a dressing. A month later he became unconscious, refused to drink, and was exceedingly nervous. Mr. R. finding him with saliva issuing from the mouth, suspected the worst, but ordered, as a temporary measure, the tepid sheet and a diaphoretic mixture. Tranquil sleep and diaphoresis followed, but about one in the morning the patient awoke screaming, had frequent convulsions, refused liquids, and foamed at the mouth. Thinking that, as a palliative, cannabis indica might be usefully employed, five minims of the tincture were given, and a short sleep followed. This dose was repeated after an interval marked by screaming fits and saliva-spit between the teeth. Deep sleep, lasting ten hours, now ensued. On awaking he recognized his mother—the first time for twenty-seven hours. His pupils were now intensely contracted. A third dose of five minims was given on the evening of the second day of medical attendance, and sleep ensued for eighteen hours. Pulse and respiration remained good all the time. From this point the progress toward recovery was steady and continuous.

VACCINE APPLIED TO SHEEP.—At a farm near Melum experiments were made yesterday by M. Pasteur in the presence of a host of specialists, on the duration of the action of anthraic vaccine as applied to sheep. It will be remembered that six months ago M. Pasteur vaccinated a number of sheep with anthratic vaccine, the immediate result being to preserve all these sheep from anthratic virus; whereas, sheep not so vaccinated succumbed within twenty-four hours to the latter. The question was how long the influence of such vaccine would last. Yesterday's experiments proved that it lasts six months, and they will be continued from month to month to ascertain the exact duration of the preservative. On Thursday, four unvaccinated sheep were inoculated with anthratic virus, as also four of the sheep vaccinated six months ago. Two of the unvaccinated sheep expired within twenty-four hours, and the other two subsequently; whereas, the sheep vaccinated six months ago admirably resisted the action of the virus. Another curious fact was ascertained. A lamb, the offspring of a vaccinated sheep, was inoculated with the virus. It expired within twenty-four hours, thus proving that the vaccine virtue is not transmitted hereditarily. The Seine-et-Marne Agricultural Society presented M. Pasteur on Thursdry with a gold medal, and a banquet was held, at which the great service rendered to agriculture by his discovery was warmly testified to.

Pleuro-pneumonia in Cattle. — The President transmitted to the Senate the report of a commission appointed by the Secretary of the Treasury to report on the lung plague of cattle, or pleuro-pneumonia. The commission, composed of Messrs. James Law, E. F. Thayer, and J. H. Sanders, have prepared an exhaustive history of the disease and the causes which led to its introduction and spread in this country. The ultimate source of lung plagues, the commission say, is not known. Up to the present time it has successfully eluded all investigations. Like small-pox, measles, the plagu e, etc., cattle lung plague is only known as it is transmitted by its own contagious products. All historic records of lung plague betray its continuous existence in different parts of Europe, where it has prevailed from time immemorial, and show further that the invasion of a new country by the pestilence has ever been the sequel of the transit of cattle from an infected region, either in the commissariat parks of a belligerent army or in the channels of an opening commerce. The report says that the pestilence began in this country in 1848, when an infected English cow was landed in Brooklyn, and has since extended about 300 miles south. The commission reach the conclusion that the unvarying absence of lung plague apart from contagion is a perfect guarantee that it can be permanently eradicated, and maintain that in every instance where a nation has stamped out the infection no new cases have appeared until there has been another importation of infected stock. Long delay in stamping out the disease in the United States means its extension to the open cattle ranges and the impossibility of stamping it out. With the near prospect of a general extension of the plague and the yearly sacrifice of tens and scores of millions of dollars to its insatiable craving, to say nothing of the continued incubus on our foreign market, to delay the work of extinction which is now in our power savors of criminality.

The Horse's Frog.—If we were to go to many a blacksmith and ask him if he did not think nature had made a mistake in putting the clumsy frog into the horse's foot, he would hardly be ready to say yes, and very likely would put on a surprised look, and perhaps explain that in some countries horses did very well without shoes, and so the frog was left to care for itself. But while not ready to take ground with you in any criticism of the plan upon which the foot is constructed, you have but to look in the corner of the shop where two horses stand newly shod; lift up their feet and observe for yourself, that if the blacksmith has not said it, the knife has said the frog is a bad thing, and must be cut away. The horses do not stand on the ground, but nearly half an inch higher, on the iron of their shoes, which takes the weight of the horse on the outer shell of the hoof. The practice is as sensible as it would be for a man who had to travel on all fours taking the weight on the nails of his fingers and toes rather than on the cushion which lies behind them. It is always the soft part—the India rubber part of the feet of animals that have such—which receives the weight, and not the shelly, hard part. We know what an elephant's foot is; it is all rubber-like. The horse has the same encased in a shell, which gives him accuracy and steadiness of movement. Now, this casing protects the frog. It grows slowly, the frog grows rapidly. The healthy foot of the colt shows a centre, if not protecting, at least level with the line of the hoof. He does not take his weight wholly on the rim of his feet. Old horses would have feet more like them if blacksmiths would allow they knew a little less than nature, and really knew enough to read her intentions.

The object in shoeing the animal, aside from the occasional one of changing its gait, is simply to prevent the wear and shattering of the outer shell, and to enable it to take a firmer hold of the ground, escaping the slipping of the unshod horn. It is an unfortunate incident of our system of shoeing that the horse is raised from the ground as a boy is when he mounts stilts. —*The Blacksmith and Wheelright.*

A Remedy for the Cattle Plague.—Mr. H. Cloete, a prominent colonist of the Cape of Good Hope, informs the Speaker of the House, through the Hon. Levi P. Morton, our Minister to France, that he and his friends are the proprietors of an effective and inexpensive remedy for pleuro-pneumonia, or lung disease, in cattle. He speaks of his method as follows:

"When one or more animals show symptoms of the disease the entire herd is put under treatment. Within ten days all those infected, however slightly, are dead. Those not infected, after being slightly indisposed for a few days, afterward become quite well, and are proof against the disease for at least seven years, and in all probability for their natural lifetime. By this mode of treatment any country can not only stamp out the disease, but insure itself against future loss. I am prepared, if the United States Government desires, to come to the States and give proof of the efficacy of this mode of treatment, and feel certain that a similar mode could be made applicable to prevent foreign cattle going into Texas from catching the Texan fever."

Mr. Cloete, who was recommended by Viscount Lyons and Lord Granville to Minister Morton, says that he is urging upon all foreign governments the consideration of his discovery.

Texas Fever, or Splenic Fever. (Also called Spanish Fever.)—Generally, after four to six weeks of co-habitation of native or Northern cattle with Texan or Southern cattle, or after exposure to the contaminating principle of this disease, an elevation of the bodily temperature of from 103° F. to 107° F., may be observed, and which is immediately followed by a general dullness and sluggishness, drooping head with hanging ears, a more or less arched back, hollow flanks, more or less stiffness of the hind quarters, and knuckling over of the hinder fetlocks. Gradually the breathing becomes quickened, and there is more or less quivering of the muscles of various parts of the body and neck. While the animals may continue to eat and ruminate to the very last, they soon lose strength, often lie down, preferably in damp or wet places, when such are readily accessible. Dropsical swellings may appear between the branches of the lower jaw, or under the chest. The muzzle is dry, and the eyes gradually become staring and glassy. The breathing becomes quicker as the disease progresses; the bowels become costive; the dung is more or less blood-stained, and towards the last the urine becomes of a deep red or nearly black color. After gradually increasing prostration and listlessness, the animal lies down motionless, outstretched in a profound stupor, in which conditions it dies slowly, and generally without a struggle. Normal condition of oxen and cows—The pulse per minute varies from 40 to 50; respiration varies from 15 to 20 per minute; average range of temperature during confinement, from 100 2-5° F. to 101 3-5° F.; when at liberty, it averages from 101° F. to 102° F.

Diarrhœa amongst Calves.—A German agricultural journal gives publicity to the experiences on the above subject of an agricultural proprietor in the Posen District, from which it would appear that, during one month, he lost from this cause fifteen calves within a few days of their birth. The symptoms were as follows: Sudden and violent diarrhœa, bad purging, general debility, followed by death on the second or third day. Those animals which survived were in a heavy, drowsy state for some time.

The examination made of the carcases of the calves which had yielded to the disease showed analogous symptoms in each case, the stomach and intestines being empty, the membrane covering the intestines partially reddened, and the lungs in several cases in a diseased condition.

The owner's suspicions lay in the direction of the feeding of the cows whose progeny was thus affected. For some months each cow had been given two pounds of rapeseed cakes, two pounds of wheat-husks, five pecks of turnips, seventeen and a half pounds of clover, nine quarts of distiller's wash —with salt as required. The rapeseed cakes he suspected were not free from admixture of mustard, and in view of the comparatively small quantity of the wash, he could only attribute the blame to these cakes. As a change of diet, he reduced the quantity of cakes to half a pound (obtained from a different source of supply), and increased the weight of wheat husks to two and a half pounds, thus effecting a reduction in the total quantity of food, as the animals were getting too fat. During the first few days after the change of diet (which took place February 23d) several calves were attacked, to which a small dose was administered three times a day, consisting of bicarbonate of soda and carbonate of magnesia. This was successful in some cases, while in others the animals had to be slaughtered. In the latter instance, however, the lungs were found not to be affected. To the calves which fell ill after March 14th, the following mixture was given: One part of carbonate of soda, one part of bicarbonate of magnesia, two parts two per cent. solution of carbolic acid, five parts water. The calves which were given thie mixture (the dose being a tablespoonful three times a day) all recovered, and afterwards remained in good health.

From the salutary action of the carbolic acid the owner of the animals concluded that parasites were the cause of the inflammation of the bowels, manifested by the redness previously alluded to as having been noticed in the coating of the intestines.

Acting upon this supposition, he caused all the walls, as well as the coverings, etc., of the cowhouse to be whitewashed, and laid down every evening plenty of disinfecting lime, so that the animals, both old aud young, would be subjected, while the doors were closed for the night, to the full influence of the carbolic acid. The result of these measures was satisfactory.

After twenty-five years' experience in weaning calves, it has been found that neither astringents, aperients, nor anti-acids have much to do in saving a calf when scouring badly. The best thing to do is never to let a healthy calf lie in the same pen as a diseased one—it is most contagious. Instant removal, warm spice gruel and cleanliness is the best cure.

A leading continental journal, in commenting upon these statements, remarks that, where cows are heavily fed, the calves are seldom in better condition than when the cows have only been kept up to an average standard of weight, experience proving that the fatter cows are, a proportionate increase of mortality and a greater liability to infection is manifested in their calves.

Lung Sickness of Cattle.—We are indebted to Mr. A. Wilkinson, of the Ottawa Estate, Natal, for the following particulars of his carbolic acid remedy, which is the only thing he has found of any use. It is well worth a trial. He writes:

"On the first appearance of the disease showing itself in a herd of cattle, give them all round a dose of carbolic acid once a day for two or three days, as a preventive to the spread of the disorder.

"The dose is thirty drops of crystalized carbolic acid in half a soda bottle of raw linseed oil (mind it is not boiled oil), twenty drops for a two-year-old, and ten drops for a yearling. When a beast has got the complaint, give the dose three times a day; when getting better, twice; and continue it some time once a day, or until all symptoms have disappeared.

"The carbolic acid must be the crystalized kind (Calvert's No. 2 acid was used), which is dissolved by placing the bottle in hot water or in the sun. It is convenient for measuring the acid to get a small phial, put the number of drops in with a minim glass, then scratch a notch on the bottle with a file.

"If the cattle could be kept in a warm shed, they would have a better chance; and if tied up, cloths dipped in common carbolic acid and water (Calvert's No. 5 quality, in 50 parts of water) hung up before their noses might be beneficial to stop the spread of the infection.

"Any cattle attacked by the disease shourd be separated from the herd at once. As soon as it can be done, a beast, when attacked, should have a blister, made of half euphorbia juice and spirits of turpentine, applied behind the shoulder-blade, low down over the lungs.

"It would be advisable at the same time, when the sickness makes its appearance in a herd, to inoculate all the cattle not attacked, by putting a seton dipped in the virus or water taken from a fresh lung, not too far gone in the disease; the seton should be inserted in the brush of the tail, and taken out the next day, and care must be taken when inoculating not to let the needle touch the bone of the tail. Should the tail swell after, lance it freely, and foment with hot water; also give a dose or two of Glauber's salts. On no account should the virus be taken from the lung of a beast which has died from lung sickness. In that case the blood would be poisoned, and probably few survive.

"This remedy, if taken in time, will, I believe, cure two out of three. Four years ago I had the lung sickness in my herd, and for the first week tried the homœopathic treatment—all died; then I tried paraffin for a week, with the same result. On commencing the carbolic acid there was very soon a change for the better, and cattle which had been standing about and not eating commenced to eat again. Half of these latter got over it, and in a fortnight were sleek and fat, the linseed oil having that effect. Should the cattle be purged lessen the quantity of oil. If this remedy had been known earlier, I believe thousands of cattle might have been saved in Natal. After the first two or three times four Kaffirs were enough to give the medicine—two to hold the horns, one to hold the head up and the tongue on one side, another to pour the dose down. Take care to hold the head up some little time until the animal has swallowed the oil."—*Land and Water.*

Wyoming Cattle Law.—A bill has recently become a law in Wyoming Territory, which undertakes to deal very strictly with cattle afflicted with contagious diseases. A Territorial Veterinarian is appointed to inspect all cattle which he may have reason to believe are affected with any contagious disease, and if found so affected, to order them killed and the carcases burned, the territory paying the owners the value of the animals destroyed. Owners of diseased cattle are forbidden to sell or give away or butcher diseased cattle. The importation and exportation of diseased cattle is forbidded, and when the cattle of any locality are found to be afflicted, none can be sent away without the order of the veterinarian. When the Governor believes that the disease is epidemic in other places outside of the territory, he may forbid the importation of cattle from such places. No person is allowed to conceal the existence of infectious diseases or obstruct the veterinarian in the performance of his duties. Heavy penalties are attached to the violation of the law. The provisions are the most thorough and rigid of any law of the kind that we have knowledge of, and evinces a determination on the part of the territorial authorities to summarily stamp out any contagious disease that may originate among or be brought to the cattle of Wyoming.

GLANDERS.—Glanders, as every one knows, is a highly contagious disorder of solipeds, and is now very prevalent in the United Kingdom. In London it is especially so, and causes great losses to owners of horses. It is readily communicable between the horse and ass species, less so between these and other species, but man is frequently infected. It is a most repulsive malady, and is incurable. Very much of our knowledge respecting it is entirely due to experiments on living animals. Not unfrequently it manifests itself in a chronic form, and with such vague symptoms (though it is, nevertheless, as contagious as if these were well marked) that the most skillful veterinary surgeon cannot tell for certain whether it is the disease or only an ordinary catarrh. If it be glanders, then to allow the animal to live is to endanger the life of every horse and man who come in contact with it; while to destroy it, if the malady is not contagious, would be cruel and unnecessary. When time is an object, or facilities for isolation are not present, then test inoculation must be resorted to. For this purpose a worthless horse, or, better still, an ass, is inoculated, and a few days suffice to decide whether glanders is present. If the result of the inoculation is affirmative, the experimental animal manifests symptoms generally at the seat of inoculation, which causes it little if any discomfort, and it is at once destroyed, as is also the suspected horse. By this precautionary procedure many horses, possibly those of an entire regiment or army corps, may be saved from peril, and human lives preserved from a loathsome and fatal disease. In elucidating the processes of disease, in framing preventive measures, in investigating the spread of contagious disorders, as well as in perfecting modes of cure, and the most humane methods of surgical operation, experiments on living creatures are absolutely necessary, for their own interests no less than for those of mankind. Veterinary medicine and surgery are based on humanity no less than on utility, and their aim is to remove or alleviate pain among the animals placed under the domini n of man. By experiments in pathology disease and mortality have been vastly diminished, and continued experiments in the same direction will cause further diminution. If mankind benefits, so do animals. A discovery which will avert disease in one will probably do so in the others; every advance of knowledge is a boon to all. To prohibit resort to experimental pathology would be at once to doom creatures which we are bound to protect to the endurance through all time of terrible suffering from diseases that might otherwise be vanquished. Abhorring cruelty in every shape, and desirous of abolishing it by every possible means, I must nevertheless deprecate the attempt to place a barrier across the path pursued in pathological investigation on animals.—*George Fleming, in Nineteenth Century.*

